# Post-mortem Rodent Scavenging Artifacts in a Cachectic Deceased Discovered in a Forest Shelter: A Case Report

**DOI:** 10.7759/cureus.117258

**Published:** 2026-09-30

**Authors:** Akhilesh Singh, Anand Kumar, Prahalad Prajapat, Nikita Sihag, Raviprakash Meshram

**Affiliations:** 1 Forensic Medicine, All India Institute of Medical Sciences, Rishikesh, Rishikesh, IND; 2 Forensic Medicine and Toxicology, All India Institute of Medical Sciences, Rishikesh, Rishikesh, IND; 3 Forensic Medicine, All India Institute of Medical Sciences, Jammu, Vijaypur, IND

**Keywords:** cachexia, forensic, post-mortem artifacts, rodent scavenging, taphonomy

## Abstract

Determining the cause and manner of death when human remains are recovered from isolated outdoor environments presents significant forensic challenges, particularly when post-mortem animal scavenging has altered the body. Rodents frequently target soft tissues, producing distinct taphonomic artifacts that can closely mimic sharp-force trauma, ante-mortem assault, or foul play. This case highlights the findings from a cachectic, unidentified adult male discovered in an abandoned forest shelter during winter (ambient temperatures between 5 °C and 16 °C), where rodent nests and rodent droppings were found. External examination revealed extensive soft-tissue destruction across the right side of the face, including the cheek, periorbital tissue, lower eyelid, nose, ear pinna, and underlying muscles and vessels. The wound exhibited pale, irregular margins marked by fine nibbling and 1-2 mm notches characteristic of rodent incisors, alongside minor passive blood pooling from post-mortem vessel disruption. The estimated post-mortem interval was 24-48 hours before post-mortem examination. Diagnosis was established on classic gross macroscopic criteria, including pale wound bases and characteristic incisor striae, supported by the complete absence of vital reaction. The cause of death was determined to be severe hypothermia secondary to extreme exogenous starvation and cachexia. Accurately diagnosing rodent scavenging relies on identifying paired tooth grooves, pale wound margins, absence of vital reaction, and ecological scene context to distinguish post-mortem scavenging artifacts from true mechanical trauma.

## Introduction

Animal scavenging presents a diagnostic challenge in forensic taphonomy and autopsy practice [1,2]. Both commensal and wild rodents, including Rattus norvegicus, Rattus rattus, and Mus musculus, routinely feed on human remains in both indoor and outdoor settings [2,3]. These animals characteristically target exposed or anatomically prominent structures where skin is thin or soft tissue is readily accessible, most commonly the eyelids, nose, ears, lips, and hands [3,4].

Typical rodent damage manifests as shallow or crater-like defects characterized by dry, pale bases and fine, parallel, or paired tooth marks along the edges ("nibbling") [4,5]. However, when scavengers bite through larger cutaneous or subcutaneous blood vessels, passive vascular leakage can cause localized blood staining [1,6]. This passive extravasation can complicate the gross examination, making a post-mortem bite closely resemble an ante-mortem injury [1,6,7]. Pathologists must accurately recognize these artifacts during gross examination and autopsy evaluation to avoid misinterpreting the manner of death or triggering false allegations of violence [7,8].

This case highlights the diagnostic challenge of post-mortem rodent scavenging on cachectic bodies in cold outdoor environments, where severe soft-tissue destruction and passive vascular leakage can closely mimic sharp-force trauma or foul play. By demonstrating how environmental cues, gross pathological criteria, and body condition interact, this report emphasizes the necessity of integrated scene analysis and meticulous autopsy evaluation in preventing false allegations of crime.

## Case presentation

An unknown adult male was discovered dead by police inside an abandoned forest shelter during the winter season. The deceased was discovered lying supine, clad in winter clothing covering the torso and extremities. Environmental recordings at the scene indicated low ambient temperatures ranging between 5 °C and 16 °C. Examination of the immediate surrounding area revealed rodent nests along with rodent droppings near the body, confirming active rodent habitation. The deceased exhibited marked cachexia. The scene was secured, and the body was subsequently transferred to the mortuary for a medico-legal autopsy to determine the cause and manner of death.

External examination revealed cachexia, characterized by loss of subcutaneous fat reserves, muscle wasting, and visible bony prominences across the thorax and extremities. Early post-mortem changes were present, moderated by the cool ambient conditions (5-16 °C), which retarded decomposition. Based on hypostasis and rigor mortis progression, the estimated post-mortem interval was approximately 24-48 hours before post-mortem examination. A prominent area of post-mortem soft-tissue destruction was localized to the right facial region. The defect involved the right cheek, periorbital tissue, lower eyelid, nasal structures, and ear pinna, along with underlying facial muscles and blood vessels, exposing the facial bones and nasal bones and cartilages. The wound margins were pale and irregular, with a classic finely serrated, nibbled pattern of tiny 1-2 mm notches characteristic of small rodent incisors. Parallel tooth grooves were visible over the exposed cartilage and bone [4,5]. Focal blood staining was present along portions of the eroded base due to passive post-mortem disruption and rupture of superficial facial vessels, without any vital active vascular response [1,6]. Gross examination revealed no underlying skull fractures, sharp-force incised margins, or ante-mortem mechanical trauma anywhere on the body (Figure 1).

**Figure 1:**
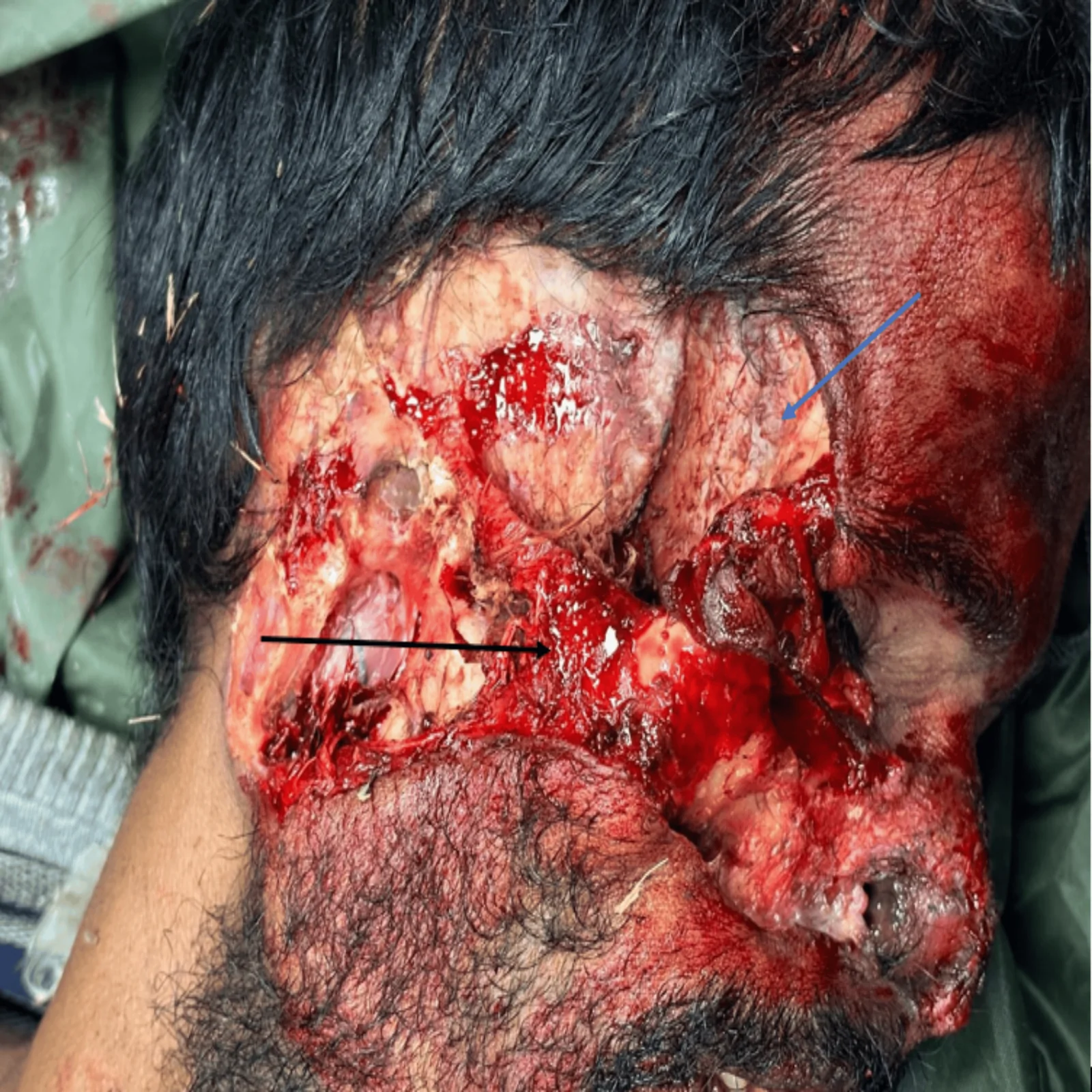
Post-mortem rodent scavenging over the right facial region. The photograph demonstrates extensive loss of skin, subcutaneous tissue, facial muscles, and blood vessels over the right side of the face, nose, lower eyelid, and ear pinna, featuring pale, irregular, nibbled margins (Blue arrow) with focal passive blood staining (Black arrow).

On internal examination, all visceral organs were atrophied, pale, and reduced in weight, except the brain. The stomach and small intestine were empty and contracted, displaying a pale, thin mucosal lining. Subcutaneous, omental, and mesenteric fat deposits were very little. The gallbladder was significantly distended and filled with thick, dark, concentrated bile, consistent with prolonged fasting or starvation. These classic macroscopic indicators confirmed severe, prolonged caloric deprivation, which significantly compromised thermoregulation prior to death. A comprehensive toxicological screen was performed and returned negative for alcohol, common drugs of abuse, and toxic substances, ruling out acute poisoning or drug-induced incapacitation.

The cause of death was certified as hypothermia secondary to severe exogenous starvation and cachexia, with the manner of death deemed natural.

## Discussion

Scavenging on human remains by rodents follows predictable anatomical and morphological patterns [2,3,8]. Rodents characteristically target exposed and prominent body areas, particularly the lips, nose, ears, eyelids, and fingertips, where the tissue is soft and easily accessible [3,5].

On gross examination, post-mortem rodent activity leaves distinct diagnostic signatures [4,5,8]:

Margin Morphology: Lesion margins appear white or distinctly pale, finely serrated, scalloped, or nibbled with a series of tiny 1-2 mm notches [4,5,8].

Tooth Mark Characteristics: Rodent dentition produces distinct parallel grooves along the tissue base and cartilage as a result of the continuous scraping action of their paired upper and lower incisors [5,8].

The environmental conditions in this case played a notable role in preserving these diagnostic features. Low winter ambient temperatures (5-16 °C) significantly slowed the rate of decomposition. Finding rodent droppings alongside nests provided ecological evidence confirming active post-mortem interference [2,3].

The primary diagnostic challenge was differentiation of post-mortem rodent activity from ante-mortem sharp-force injury, blunt impact, or live animal attack [1,7]. On gross examination, the wound edges and underlying base were pale and completely lacked vital blood infiltration, marginal tissue bruising, or active extravasation, proving the defect was inflicted after systemic circulation had ceased [1,6,7]. Although focal blood staining was observed along the defect base, this resulted from passive post-mortem blood pooling due to mechanical disruption of facial vessels by scavengers [1,6].

While rodents typically target thin periorbital skin and eyelids, extreme thinness of tissue in cachectic individuals allows rodents easier access to deeper ocular structures, explaining the extensive destruction of the eyeball and surrounding musculature [3,4]. Rodent scavenging can be differentiated from canine or feline scavenging, which typically causes large, ragged soft-tissue tearing, extensive crushing fractures, and puncture wounds from large canine teeth [1,8]. Insect activity (such as blowfly larvae) usually produces rounded, fluid-filled, or "punched-out" skin defects without the fine, linear tooth grooves characteristic of rodent incisors [3].

## Conclusions

This case underscores the importance of recognizing rodent scavenging artifacts in bodies. Meticulous gross evaluation of defect margins, specifically documenting pale bases, the absence of vital blood infiltration, 1-2 mm scalloped notches, and characteristic parallel incisor grooves, supplemented by scene investigation and autopsy evaluation, is essential to prevent misinterpreting post-mortem animal predation as ante-mortem assault or foul play.

While estimating the post-mortem interval in low ambient temperatures carries inherent margins of error, combining scene evidence with post-mortem findings provides a definitive diagnostic path. The total absence of defensive injuries, vital vascular reaction, or underlying skeletal fractures, coupled with environmental context and clear macroscopic proof of extreme starvation, definitively excluded ante-mortem violence, mechanical trauma, or accidental injury, confirming a natural manner of death secondary to severe hypothermia.
